# Influence of white matter lesions on the prognosis of acute cardioembolic stroke without reperfusion therapy

**DOI:** 10.1186/s12883-021-02372-9

**Published:** 2021-09-18

**Authors:** Yikun Guo, Zhuoyou Chen, Qian Wang, Min Zhang, Guanzhong Dong, Wenying Zou, Tian Yao, Yun Xu

**Affiliations:** 1 Department of Neurology, Nanjing Drum Tower Hospital Clinical College of Nanjing Medical University, 321# Middle Zhongshan Road, Jiangsu Province 210008 Nanjing, China; 2 Department of Neurology, The Affiliated Changzhou No.2 People’s Hospital of Nanjing Medical University, 213000 Changzhou, China

**Keywords:** Acute cardioembolic stroke, White matter lesions, Microcirculation disorder, Functional outcome

## Abstract

**Background:**

Few studies have investigated the influence of white matter lesions (WMLs) on the prognosis of acute cardioembolic stroke (CES). We aimed to explore the role of WMLs in predicting 3-month prognosis of CES without reperfusion therapy.

**Methods:**

A number of 251 acute CES patients without reperfusion therapy at a single center were retrospectively recruited. The severity of WMLs was evaluated by Fazekas scale and patients were divided into mild WMLs group (188 cases, Fazekas ≤ 2 points) and moderate to severe WMLs group (63 cases, Fazekas ≥ 3 points) accordingly. General data and clinical features of the two groups were compared. Functional outcomes of patients were followed up for 3 months using the modified Rankin scale (mRS) and patients were divided into poor outcome group (mRS ≥ 3) and favorable outcome group (mRS ≤ 2). The effect of WMLs on the prognosis was identified by binary logistic regression.

**Results:**

Patients in moderate to severe WMLs group were older (*P* < 0.001). Also, they had higher baseline National Institutes of Health Stroke Scale (NIHSS) score (*P* < 0.001) and elevated incidence of asymptomatic cerebral hemorrhage (*P* = 0.040) and stroke associated pneumonia (*P* = 0.001) than those in mild WMLs group. At 3 months, there were 100 cases in the poor outcome group. Patients in poor outcome group had higher baseline NIHSS score, increased proportion of moderate to severe WMLs, and elevated incidence of stroke associated pneumonia than those in favorable outcome group (*P* < 0.001). Binary logistic regression analysis showed that moderate to severe WMLs (odds ratio [OR] = 4.105, 95 % confidence interval [CI] = 1.447–11.646), baseline NIHSS score (OR = 1.368, 95 % CI = 1.240–1.511), and stroke-associated pneumonia (OR = 4.840, 95 %CI = 1.889–12.400) were independent risk factors for poor outcome.

**Conclusions:**

Moderate to severe WMLs is an independent risk factor for prognosis of CES patients without reperfusion therapy.

## Background

White matter lesions (WMLs) characterized by bilateral, mostly symmetrical hyperintensities on T2-weighted and fluid attenuated inversion recovery (FLAIR) MRI sequences are an imaging manifestation of cerebral small vessel disease and an important marker of cerebral microcirculation disorder [1, 2]. Studies have found that WMLs are a risk factor for stroke, dementia and death in the general and high-risk population [3]. In addition, they can determine the severity and hinder the neurological recovery of stroke [4–6]. Cardioembolic stroke (CES) accounts for around 15–30 % of ischemic stroke [7]. As thrombus from heart suddenly blocks the intracranial artery, the status of cerebral microcirculation may affect the establishment of secondary and tertiary collateral vessels, and thus influence the outcomes of CES patients.

However, few studies have focused on the relationship between WMLs and the functional outcomes of CES patients [8, 9]. This study aimed to investigate the effect of WMLs on the 3-month prognosis of CES patients without reperfusion therapy.

### Methods

For this single-center, retrospective, observational study, we retrieved data from the stroke center database of Changzhou NO.2 People’s Hospital, Jiangsu Province, China. This study was approved by the Clinical Research Ethics Committee of the Affiliated Changzhou No.2 People’s Hospital of Nanjing Medical University (2018KY032-01). Verbal consents were obtained from all the patients or their legally authorized representatives.

### Participants

Consecutive patients with first-ever acute CES within 48 h of stroke onset from June 2014 to September 2019 were included. All patients had known or newly-diagnosed non-valvular atrial fibrillation, and stroke events were confirmed by magnetic resonance imaging (MRI) or computerized tomography (CT). Other inclusion criteria were: (1) 18 ≤ age ≤ 80 years, (2) anterior circulation infarction, and (3) involvement of at least one side of the middle cerebral artery. Patients were excluded if they had reperfusion therapy or had no brain MRI/magnetic resonance angiography (MRA) or CT/computerized tomography angiography (CTA) which would be essential to estimate the stroke subtype.

### Diagnosis of cardioembolic stroke

Essential tests like electrocardiogram, transthoracic echocardiography, cervical vascular ultrasound and cranial CTA/MRA were routinely performed. Thus, the known or newly-diagnosed non-valvular atrial fibrillation was confirmed and the stroke subtype of CES defined by the Trial of Org 10,172 in Acute Stroke Treatment (TOAST-criteria) was identified [10].

### Grading of white matter lesions

**Fig. 1:**
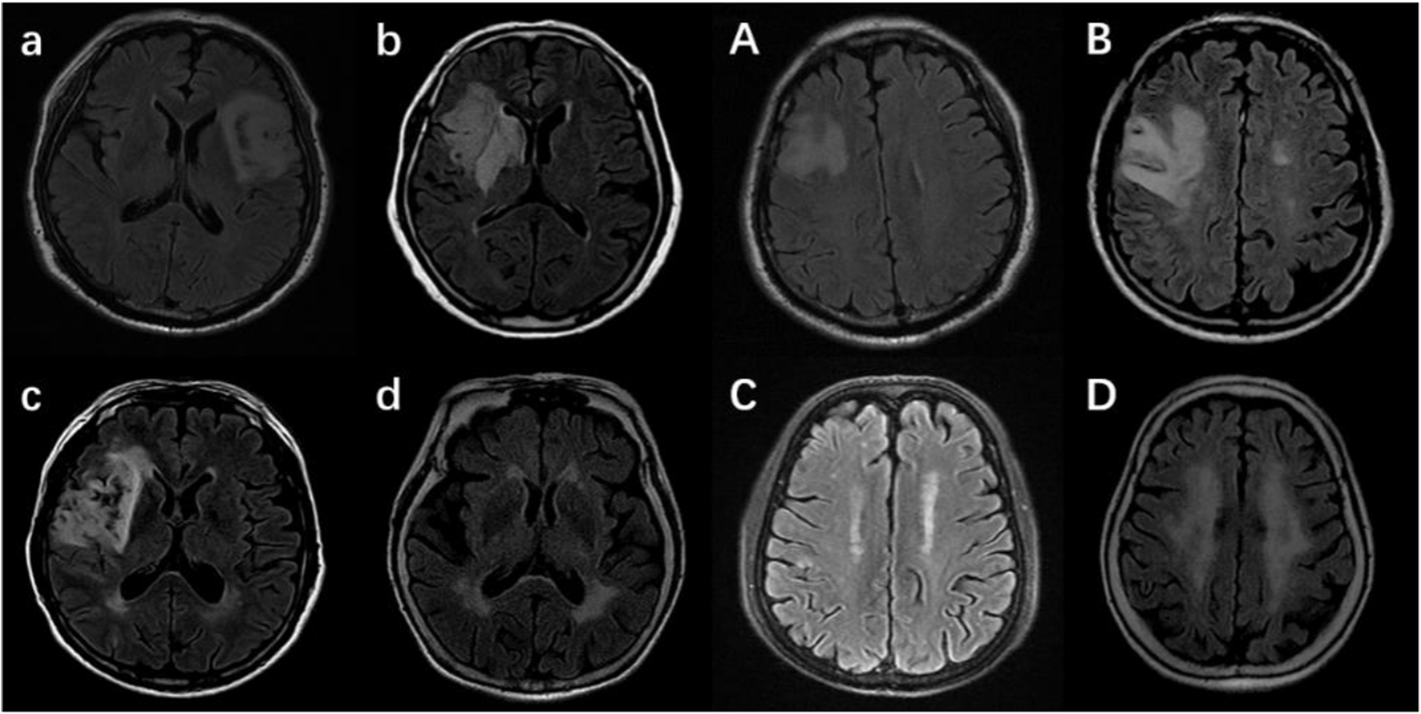
Grading of white matter lesions (WMLs) by the Fazekas scale. a, b, c, d indicates 0, 1, 2, and 3 for Fazekas scale score of periventricular WMLs. A, B, C, D indicates 0, 1, 2, and 3 for Fazekas scale score of deep WMLs.

The extent of white matter lesions (WMLs) was graded by the Fazekas scale according to the non-contrast CT scans or FLAIR MRI imaging [11]. Periventricular and deep WMLs were rated separately. Periventricular WMLs were scored as follows: 0 = absence, 1 = caps or pencil-thin lining, 2 = smooth halo, and 3 = irregular periventricular hyperintensities extending into the deep white matter. Deep WMLs were scored as follows: 0 = absence, 1 = punctuate foci, 2 = beginning confluence of foci, and 3 = large confluent areas. A total Fazekas WMLs score, ranging from 0 to 6, was obtained by summing the periventricular and deep white matter scores. Mild WMLs was defined as Fazekas score ≤ 2, and moderate to severe WMLs as Fazekas score ≥ 3 (Fig. 1).

### Clinical evaluation and functional follow-up

Baseline neurological deficit was assessed with the National Institutes of Health Stroke Scale (NIHSS) score.

An intracranial hemorrhage was defined as symptomatic (sICH) if patient had clinical deterioration causing an increasement in the NIHSS score by ≥ 4 points and as non-symptomatic (nsICH) otherwise [12].

Stroke-associated pneumonia (SAP) was diagnosed in the presence of fever, purulent sputum, abnormal respiratory examination, and pathologic chest X-ray findings and/or leukocytosis or leukopenia (white blood cells count ≥ 10 × 10^9^ or ≤ 4 × 10^9^/L, respectively) [13].

Functional outcome was evaluated by the modified Rankin Scale (mRS) via telephone or a face-to-face manner at 3 months after stroke onset. A doctor and a trained nurse separately judged the function status of each patient and then achieved a consistent result. Accordingly, patients were divided into poor outcome group (mRS ≥ 3) and favorable outcome group (mRS ≤ 2).

### Statistical analysis

Continuous variables were presented as mean ± standard deviation (SD) or median (interquartile range, IQR) and compared between groups using Student’s t-test or Kruskal-Wallis *H* test. Categorical variables were expressed as percentage and compared between groups with Pearson’s χ2 test. Association between WMLs and 3-month functional outcome was analyzed using binary logistic regression. The results are shown as odds ratio (OR) and 95 % confidence intervals (CI). A two-tailed *P* < 0.05 was considered statistically significant. All statistical analysis was performed on SPSS 22.0 (IBM Corp., Chicago, IL, USA).

## Results

### Baseline information

Among the 292 patients who initially met the inclusion criteria, 41 patients were lost to follow-up. Thus, a total of 251 patients were included for final analysis, of whom 245 (97.6 %) underwent at least one MRI scan. The median age was 73 years (IQR 67, 77) and the median admission NIHSS score was 7 (IQR 3,14). Sixty-three participants (25.1 %) had moderate to severe WMLs (Table 1).

**Table 1:** Baseline information

Variable	*n* = 251
Age, y, median (IQR)	73 (67, 77)
Male sex, n (%)	138 (55)
Smoking, n (%)	62 (24.7)
Hypertension, n (%)	196 (78.1)
Diabetes mellitus, n (%)	52 (20.7)
Hyperlipidemia, n (%)	43 (17.1)
Congestive heart failure, n (%)	52 (20.7)
Baseline SBP (mm Hg, ‾x ± s)	146.5 ± 19.8
Baseline DBP (mm Hg, ‾x ± s)	86.1 ± 13.2
Baseline NIHSS score, median (IQR)	7 (3, 14)
Moderate-severe WMLs, n (%)	63 (25.1)
sICH, n (%)	27 (10.8)
nsICH, n (%)	46 (18.3)
SAP, n (%)	79 (31.5)
Anticoagulant therapy, n (%)	142 (56.6)
Stroke recurrence	8 (3.2)
Laboratory findings, mmol/l	
Total cholesterol (‾x ± s)	4.1 ± 0.8
Triglyceride (‾x ± s)	1.2 ± 0.8
LDL-C (‾x ± s)	2.2 ± 0.7
Fasting glucose (‾x ± s)	6.3 ± 2.2

Abbreviations: *IQR* interquartile range; *SBP* systolic blood pressure; *DBP* diastolic blood pressure; *NIHSS* National Institutes of Health Stroke Scale; *WMLs* white matter lesions; *sICH* symptomatic intracerebral hemorrhage; *nsICH* non-symptomatic intracerebral hemorrhage; *SAP* stroke-associated pneumonia; *LDL-C* low-density lipoprotein cholesterol

### Differences of variables between the two WMLs groups

Sixty-three patients were classified into the moderate to severe WMLs group, and 188 patients were included in the mild WMLs group. Patients in the moderate to severe WMLs group were older (P < 0.005), had higher baseline NIHSS score (*P* < 0.005) and had higher proportion of hypertension and congestive heart failure (P < 0.005). Incidences of SAP (47.6 % vs. 26.1 %, p = 0.001) and nsICH (27.0 vs. 15.4 %, *p* = 0.040) in moderate to severe WMLs group were significantly higher than those in mild group. However, there was no significant difference in the incidence of sICH (15.9 vs. 9.0 %, *p* = 0.130) between the two groups (Table 2).

**Table 2:** Differences of variables between the two WMLs groups

Variable	moderate to severe WMLs group	mild WMLs group	Test Value	*P* Value
	**(*n* = 63)**	**(*n* = 188)**		
Age, y, median (IQR)	77 (74,79)	71 (66,75)	6.241	**< 0.001**
Male, n (%)	36 (57.1)	102 (54.3)	0.159	0.690
Smoking, n (%)	14 (22.2)	48 (25.5)	0.278	0.598
Hypertension, n (%)	60 (95.2)	136 (72.3)	14.459	**< 0.001**
Diabetes mellitus, n (%)	17 (27.0)	35 (18.6)	2.011	0.156
Hyperlipidemia, n (%)	12 (19.0)	31 (16.5)	0.218	0.641
Congestive heart failure, n (%)	23 (36.5)	29 (15.4)	12.769	**< 0.001**
Baseline SBP (mm Hg, ‾x ± s)	152.5 ± 21.0	143.7 ± 21.3	2.870	**0.005**
Baseline DBP (mm Hg, ‾x ± s)	88.4 ± 14.0	85.4 ± 12.9	1.490	0.139
Baseline NIHSS score, median (IQR)	11 (6,16)	6 (2,13)	3.514	**< 0.001**
Fazekas score, median (IQR)	3 (3,4)	2 (1,2)	12.492	**< 0.001**
sICH, n (%)	10 (15.9)	17 (9.0)	2.293	0.130
nsICH, n (%)	17 (27.0)	29 (15.4)	4.212	**0.040**
SAP,n (%)	30 (47.6)	49 (26.1)	10.165	**0.001**
Anticoagulant therapy, n (%)	32 (50.8)	110 (58.5)	1.144	0.285
Stroke recurrence, n (%)	3 (4.8)	5 (2.7)	0.166	0.411
Laboratory findings, mmol/l				
Total cholesterol (‾x ± s)	4.1 ± 0.8	4.1 ± 0.9	0.146	0.884
Triglyceride (‾x ± s)	1.1 ± 0.5	1.2 ± 0.9	-1.234	0.219
LDL-C (‾x ± s)	2.3 ± 0.7	2.2 ± 0.7	1.232	0.221
Fasting glucose (‾x ± s)	6.3 ± 2.4	6.2 ± 2.2	0.314	0.754

### Differences of risk factors and clinical findings between the two outcome groups

Totally, 100 (39.8 %) patients were defined as having poor outcome. Compared with patients in the favorable outcome group, those who had poor outcome had much severe WMLs (moderate-severe WMLs: 39.0 % vs. 15.9 %, *P* < 0.001) and higher baseline NIHSS score (*P* < 0.001). Also, they were older (*P* = 0.002)). Additionally, they had significantly higher rates of sICH (17.0 % vs. 6.6 %, *P* = 0.009), nsICH (30 % vs. 10.6 %, *P* < 0.001) and SAP (66.0 % vs. 8.6 %, *P* < 0.001) (Table 3)

**Table 3:** Differences of risk factors and clinical findings between the two outcome groups

Variable	Poor outcome	Favorable outcome	Test Value	*P* Value
	(*n* = 100)	(*n* = 151)		
Age, y, median (IQR)	75 (70, 78.75)	72 (66, 75)	3.658^a^	**< 0.001**
Male, n (%)	58 (58.0)	80 (53.0)	0.612^c^	0.434
Smoking, n (%)	24 (24.0)	38 (25.2)	0.044^c^	0.834
Hypertension, n (%)	83 (83.0)	113 (74.8)	2.344^c^	0.126
Diabetes mellitus, n (%)	23 (23.0)	29 (19.2)	0.527^c^	0.468
Hyperlipidemia, n (%)	14 (14.0)	29 (19.2)	1.148^c^	0.284
Congestive heart failure, n (%)	30 (30.0)	22 (14.6)	8.721^c^	**0.003**
Baseline SBP (mm Hg, ‾x ± s)	149.6 ± 24.5	143.5 ± 19.0	2.113^b^	**0.036**
Baseline DBP (mm Hg, ‾x ± s)	87.9 ± 14.1	85.0 ± 12.6	1.707^b^	0.089
Baseline NIHSS score, median (IQR)	15 (11, 19)	4 (2, 7)	11.150^a^	**< 0.001**
Moderate-severe LA, n (%)	39 (39.0)	24 (15.9)	17.084^c^	**< 0.001**
sICH, n (%)	17 (17.0)	10 (6.6)	6.749^c^	**0.009**
nsICH, n (%)	30 (30.0)	16 (10.6)	15.133^c^	**< 0.001**
SAP, n (%)	66 (66.0)	13 (8.6)	63.224^c^	**< 0.001**
Anticoagulant therapy, n (%)	56 (56.0)	64 (42.4)	0.022^c^	0.881
Stroke recurrence, n (%)	4 (4.0)	4 (2.6)	0.004^c^	0.953
Laboratory findings, mmol/l				
Total cholesterol (‾x ± s)	4.1 ± 0.9	4.1 ± 0.8	0.703^b^	0.482
Triglyceride (‾x ± s)	1.0 ± 0.4	1.3 ± 0.9	-2.843^b^	**0.005**
LDL-C (‾x ± s)	2.3 ± 0.7	2.2 ± 0.7	1.412^b^	0.152
Fasting glucose (‾x ± s)	7.0 ± 2.7	5.8 ± 1.7	3.844^b^	**< 0.001**

^a^ Kruskal-Wallis *H* test; ^b^*t* test; ^c^χ2 test.

### Impact of moderate to severe WMLs on the 3-month prognosis in the regression model

After adjustment for parameters with p values < 0.05 in univariate analysis, multivariate analysis using binary logistic regression with enter method was performed. The results showed that moderate to severe WMLs (OR = 4.105, 95 %CI = 1.447–11.646, *P* = 0.008), baseline NIHSS (OR = 1.368, 95 %CI = 1.240–1.511, *P* < 0.001), SAP (OR = 4.840, 95 %CI = 1.889–12.400, *P* = 0.001) and nsICH (OR = 3.751, 95 %CI = 1.234–11.399, *P* = 0.020) were independent risk factors for a poor prognosis at 3 months (Table 4).

**Table 4:** Influence of moderate to severe WMLs on the 3-month prognosis

Variable	OR	95 %CI	*P* Value
Moderate-severe WMLs	4.105	1.447–11.646	**0.008**
Baseline NIHSS score	1.368	1.240–1.511	**< 0.001**
nsICH	3.751	1.234–11.399	**0.020**
SAP	4.840	1.889–12.400	**0.001**

## Discussion

The present study described the overall distribution of WMLs in CES patients and found that moderate to severe WMLs was an independent risk factor for poor prognosis at 3 months in such patients without reperfusion therapy.

Researches have showed that WMLs are widely distributed in the elderly and stroke patients and are an indicator of cerebral microcirculation disturbance [2, 14]. For example, a study scanned 26 cognitively normal elderly subjects with arterial spin labeling (ASL) sequence, and found that the volume of periventricular WMLs was positively correlated with decreased regional cortical blood flow [15]. Another study indicated that the severity of WMLs was negatively associated with leptomeningeal collateral circulation rating in patients with acute large artery occlusion, which further suggested that WMLs could disrupt collateral circulation compensation and decrease regional cerebral blood flow [16]. In our study, the incidences of sICH and nsICH in moderate to severe WMLs group were higher than those in mild WMLs group, which may be related to the more serious damage of microcirculation caused by extended WMLs.

The essence of CES is acute cerebral vascular occlusion resulted in cardiac embolus shedding. In this instance, the survival of ischemic brain tissue largely depends on the collateral compensation, which is partly conditioned by microcirculation. As a result, the presence of moderate to severe WMLs may indicate insufficient collateral circulation compensation and unfavorable prognosis with respect to neurological recovery [16]. In our study, moderate to severe WMLs was an independent risk factor for 3 months prognosis of CES patients, which is consistent with the previous research results. However, one study has shown that WMLs affects the outcomes of different stroke types differently. In that study, WMLs volume was positively correlated with the probability of poor prognosis in patients with stroke subtype of large artery atherosclerosis and small artery occlusion but not in patients with CES [17]. We speculate that the different research conclusions may be due to unequal neurological deficit, which is the key determinant of stroke outcome [18].

WMLs may affect the prognosis of stroke through other mechanisms [19]. First of all, WMLs could damage the microstructure of white matter tissue and disrupt the brain neural connections and functional network, thus impairing the plasticity and compensatory mechanisms and slow down the brain’s recovery from stroke [20]. Besides, WMLs could disrupt motor/cognitive networks that are important for learning and neurorehabilitation [21, 22].

There are many other factors affecting the outcome of stroke patients, such as the baseline NIHSS score, SAP and age. In our study, patients with higher NIHSS score have much higher risk of developing SAP, which is consistent with previous research [23]. We speculate that the possible reasons are the widespread disturbance of consciousness, indwelling gastric cannula, paralysis and assisted breathing in patients with much severer neurological deficits.

Our study has some limitations. First, this retrospective study adopted a single center database and had a relatively small sample size of CES patients, which may increase the possibility of selection bias. Second, patients who died rapidly after admission were excluded, which may overestimate the impact of WMLs on prognosis.

In conclusion, moderate to severe WMLs was an independent risk factor for poor outcome of CES patients without reperfusion therapy. Measures should be taken on the evaluation and management of WMLs in CES patients.

## Data Availability

The datasets during and/or analyzed during the current study available from the corresponding author on reasonable request.

## Abbreviations

ASL: Arterial spin labeling
CES: Cardioembolic stroke
CI: Confidence interval
CT: Computerized tomography
CTA: Computerized tomography angiography
FLAIR: Fluid attenuated inversion recovery
M(IQR): Median (interquartile range)
MRI: Magnetic resonance imaging
MRA: Magnetic resonance angiography
mRS: Modified Rankin scale
NIHSS: National Institutes of Health Stroke Scale
nsICH: Non-symptomatic intracranial hemorrhage
sICH: Symptomatic intracranial hemorrhage
OR: Odds ratio
SAP: Stroke-associated pneumonia
SD: Standard deviation
WMLs: White matter lesions
